# Effects of pulmonary endarterectomy and balloon pulmonary angioplasty in older adults with chronic thromboembolic pulmonary hypertension: A sub-analysis of the CTEPH AC registry

**DOI:** 10.1016/j.ijcha.2025.101751

**Published:** 2025-07-12

**Authors:** Jun Yamashita, Hitoshi Ogino, Kohei Masaki, Yu Taniguchi, Shiro Adachi, Takumi Inami, Kazuya Hosokawa, Ichizo Tsujino, Nobuhiro Yaoita, Masaru Hatano, Nobutaka Ikeda, Nobuhiro Tanabe, Hiroto Shimokawahara, Kayoko Kubota, Ayako Shigeta, Koshin Horimoto, Yoshito Ogihara, Yoshihiro Dohi, Takahiro Hiraide, Takashi Kawakami, Hidekazu Ikemiyagi, Yuichi Tamura, Yoshihiro Fukumoto, Kohtaro Abe, on behalf of the CTEPH AC Registry Study Group

**Affiliations:** a Department of Cardiology, Tokyo Medical University, Tokyo, Japan; b Department of Cardiovascular Surgery, Tokyo Medical University, Tokyo, Japan; c Department of Cardiovascular Medicine, Kyushu University Graduate School of Medical Sciences, Fukuoka, Japan; d Department of Cardiovascular Medicine, Kobe University Graduate School of Medicine, Kobe, Japan; e Department of Cardiology, Nagoya University Hospital, Nagoya, Japan; f Department of Cardiovascular Medicine, Kyorin University School of Medicine, Mitaka, Japan; g Department of Respirology, Faculty of Medicine, Graduate School of Medicine, Hokkaido University, Sapporo, Japan; h Department of Cardiovascular Medicine, Tohoku University Hospital, Sendai, Japan; i Department of Cardiovascular Medicine, The University of Tokyo, Graduate School of Medicine, Tokyo, Japan; j Division of Cardiovascular Medicine, Toho University Medical Center Ohashi Hospital, Tokyo, Japan; k Pulmonary Hypertension Center, Chibaken Saiseikai Narashino Hospital, Narashino, Japan; l Department of Cardiology, NHO Okayama Medical Center, Okayama, Japan; m Department of Cardiovascular Medicine and Hypertension, Kagoshima University Graduate School of Medical and Dental Sciences, Kagoshima, Japan; n Department of Respirology, Chiba University Graduate School of Medicine, Chiba, Japan; o Department of Cardiovascular Medicine, Matsuyama Red Cross Hospital, Matsuyama, Japan; p Department of Cardiology and Nephrology, Mie University Graduate School of Medicine, Tsu, Japan; q Department of Cardiovascular Medicine, Kure Kyosai Hospital, Kure, Japan; r Department of Cardiology, Keio University School of Medicine, Tokyo, Japan; s Division of Cardiology, Department of Medicine, Showa University School of Medicine, Tokyo, Japan; t Department of Cardiovascular Medicine, Nephrology and Neurology, University of the Ryukyus Graduate School of Medicine, Okinawa, Japan; u Pulmonary Hypertension Center, International University of Health and Welfare Mita Hospital, Tokyo, Japan; v Division of Cardiovascular Medicine, Department of Internal Medicine, Kurume University School of Medicine, Kurume, Japan

**Keywords:** Chronic thromboembolic pulmonary hypertension, Pulmonary endarterectomy, Balloon pulmonary angioplasty, Older adults

## Abstract

**Background:**

Invasive treatments for chronic thromboembolic pulmonary hypertension (CTEPH) include pulmonary endarterectomy (PEA) and balloon pulmonary angioplasty (BPA). Both PEA and BPA demonstrate significant benefits for symptom relief, hemodynamic improvement, and overall patient outcomes. However, evidence of their efficacy in older adults remains limited. This study aimed to evaluate treatment efficacy—including exercise tolerance, hemodynamics, functional status—and clinical outcomes in CTEPH patients aged ≥70 years who underwent PEA or BPA.

**Methods:**

Among 1,527 patients in the CTEPH AntiCoagulants Registry, we selected those aged 70 years or older, including 25 who underwent PEA during follow-up and 210 who received BPA. We evaluated the World Health Organization functional class (WHO FC), a measure of subjective symptoms, along with exercise capacity, hemodynamic parameters, and clinical outcomes.

**Results:**

At baseline, BPA patients were older (P = 0.003) and tended to have lower body weight (P = 0.069) than those undergoing PEA. At a median follow-up of ∼2 years, BPA significantly improved six-minute walk distance and cardiac index, and reduced mean pulmonary artery pressure and pulmonary vascular resistance, with effects comparable to PEA after adjusting for age, sex, and body mass index (all adjusted P > 0.10). However, improvement in WHO FC was more frequent with BPA and became significant after covariate adjustment (adjusted P = 0.035). Adverse event rates were infrequent and similar between the two groups (all P > 0.10).

**Conclusions:**

In adults aged ≥ 70 years with CTEPH, BPA improved hemodynamics, exercise capacity, and outcomes comparable to PEA, but with greater WHO FC improvement. Further randomized trials in older adults are warranted.

**Clinical trial registration number:**

UMIN Clinical Trials Registry (identifier UMIN 000033784).

## Highlights

• CTEPH often requires invasive interventions.
• PEA and BPA are both established treatments for CTEPH.
• We investigated the efficacy of PEA and BPA in older CTEPH patients (≥70 years).
• Overall clinical outcomes were generally comparable between PEA and BPA.
• WHO functional class improved more frequently with BPA.

## 1 Introduction

Chronic thromboembolic pulmonary hypertension (CTEPH) is a disease characterized by pulmonary hypertension (PH) resulting from the chronic narrowing or occlusion of pulmonary arteries due to organized thrombi [1,2]. Although CTEPH was previously associated with poor outcomes, recent advancements in various treatments have significantly improved its prognosis [[3], [4], [5]]. Pulmonary endarterectomy (PEA) remains the standard treatment for CTEPH, with numerous studies reporting significant symptom improvement and favourable long-term outcomes. However, PEA is a highly invasive procedure that involves a median sternotomy incision and requires hypothermic cardiopulmonary bypass at temperatures below 20 °C. In Japan, balloon pulmonary angioplasty (BPA) is widely adopted, with outcomes comparable to those of PEA [[6], [7], [8], [9]]. As a catheter intervention, BPA is minimally invasive; however, it is associated with a relatively high rate of complications, including pulmonary hemorrhage [6]. In recent years, Japan's aging population has grown significantly, with clinical survey data from the Ministry of Health, Labour, and Welfare indicating that incidence peaks among individuals in their 70 s and continues to rise among those in their 80 s. However, the impact of invasive treatments such as PEA and BPA on symptoms, physical activity, and long-term outcomes in older adults with CTEPH remains insufficiently studied. This study aimed to assess the characteristics, treatment efficacy, and long-term outcomes of patients with CTEPH aged 70 years or older who underwent either PEA or BPA.

## 2 Methods

### 2.1 Study design

This study included patients with CTEPH enrolled in the CTEPH AntiCoagulants Registry (CTEPH AC Registry), a nationwide, multicenter, prospective, observational study involving 37 specialized CTEPH treatment centres (https://cteph-registry.jp/en/). The registry was launched in August 2018 and is ongoing. The study adhered to the ethical principles outlined in the World Medical Association Declaration of Helsinki for research involving human subjects. It received centralized approval from the Ethics Committee of the Graduate School and Faculty of Medicine, Kyoto University (R1919-16), and was also approved by the institutional review board of each participating centre. Registration in the open-access database UMIN Clinical Trials Registry was completed under the identifier UMIN 000033784. After obtaining written informed consent, each participant was assigned a unique patient identification number to maintain confidentiality. The study enrollment period spanned from August 2018 to December 2023. Investigators recorded the most recent data available within 12 months prior to enrollment as baseline data. Mandatory follow-up data entry was scheduled annually in November. Additional data were collected when predefined clinical worsening events of CTEPH occurred or when right heart catheterization was performed. Patients with no follow-up data for over one year were considered to have missing follow-up data.

### 2.2 Study population

Among the patients registered in the CTEPH AC Registry, those who had already undergone BPA or PEA at the time of registration were excluded. In addition, patients without follow-up data, those with an insufficient follow-up period, and those who did not undergo either PEA or BPA during the follow-up period were excluded. Among the remaining patients who underwent PEA or BPA during follow-up, individuals aged <70 years were excluded. The final study population consisted of patients aged ≥70 years, who were then classified into two groups according to the treatment received. Treatment allocation was based on clinical judgment at each participating centre**.** The diagnosis of CTEPH was confirmed using at least two imaging modalities—ventilation-perfusion scan, computed tomography pulmonary angiography, or catheter-based pulmonary angiography—in conjunction with hemodynamic criteria: resting mean pulmonary artery pressure (mean PAP) ≥25 mmHg and pulmonary artery wedge pressure ≤15 mmHg [4].

### 2.3 Data collection and analyses

Data for eligible patients were collected by investigators at each institution and compared between the two groups. These data included baseline demographics (age, sex, height, body weight, and body mass index); disease severity indices (World Health Organization functional class [WHO FC], 6-minute walk distance [6MWD], mean PAP, pulmonary vascular resistance [PVR], cardiac index [CI], brain natriuretic peptide [BNP], mixed venous oxygen saturation [SvO2], estimated glomerular filtration rate [eGFR]), comorbidities and past medical histories (history of acute venous thromboembolism [VTE], intravenous device use, active cancer or history of cancer, varicose veins, thyroid disease, chronic obstructive pulmonary disease, interstitial lung disease, hemiplegia or paraplegia, inflammatory bowel disease, history of stroke or transient ischemic attack, ovarian or uterine disease, and use of antipsychotics or prednisolone); and medication status (including use of anticoagulants and pulmonary vasodilators). Additionally, disease severity indices at the most recent follow-up after PEA or BPA were compared with baseline values. Throughout the follow-up period, the incidence of adverse events (all-cause death, lung transplantation, use of prostaglandin I2 infusion, performance of rescue interventions such as BPA and PEA, worsening PH, major bleeding, minor bleeding, and recurrence of VTE) was also compared between the two groups.

### 2.4 Statistical analyses

Continuous variables are presented as mean and standard deviation, except for the follow-up period and BNP values, which are presented as median and interquartile range (25th and 75th percentiles). Categorical variables are presented as numbers (percentages). Differences between the two groups were analysed using the unpaired *t*-test for parametric variables, the Mann–Whitney *U* test for nonparametric variables, and the chi-squared test for categorical variables. Comparisons of disease severity indices between baseline and follow-up in the PEA and BPA treatment groups were analyzed using the paired samples *t*-test for parametric variables and Wilcoxon's signed-rank test for nonparametric variables. Changes in medications from baseline to follow-up in both treatment groups were analysed using McNemar’s test. Additionally, analysis of covariance was used to assess the association between changes in clinical parameters (i.e., 6MWD and mean PAP) and treatment strategies (BPA or PEA). A linear regression model was fitted, including variables indicating treatment group and baseline values of the respective clinical parameters, whereas categorical outcomes were assessed using a logistic regression model. The multivariable model included age, sex, and body mass index as covariates. Given the limited number of patients in the PEA group (n = 25), propensity score matching and inverse probability weighting were not applied, as these methods could have led to unstable estimates or data loss. Therefore, multivariable regression models were used as the most statistically appropriate approach to control for baseline confounding [10]. The Kaplan–Meier method (log-rank test) was used to compare adverse events between the two groups. A multivariable logistic regression model was employed to assess the associations between treatment strategy and adverse events, adjusting for the above covariates.

A *p*-value of <0.05 was considered statistically significant. Imputation of missing data was not performed. Statistical analyses were conducted using SPSS (IBM SPSS Inc., Chicago, IL, USA) and R version 4.2.2 (R Development Core Team, Vienna, Austria).

## 3 Results

### 3.1 Study population

**Fig. 1:**
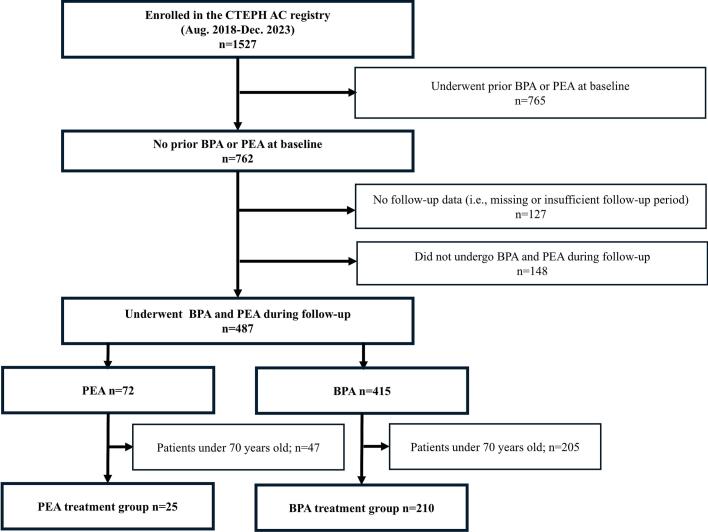
**Patient flowchart.** CTEPH, chronic thromboembolic pulmonary hypertension; BPA, balloon pulmonary angioplasty; PEA, pulmonary endarterectomy.

Between August 2018 and December 2023, 1,527 patients from 37 centres were enrolled in the CTEPH AC Registry. Among these patients, 762 had not received reperfusion therapy, such as PEA or BPA, at baseline. After excluding 127 patients without follow-up data and 148 patients for whom PEA or BPA was not planned, 487 patients were classified as having undergone PEA or BPA during the follow-up. Among them, 72 patients underwent PEA at 15 centres, while 415 patients underwent BPA in 25 centres. After excluding individuals under the age of 70, a total of 25 patients aged ≥70 were included in the PEA treatment group (12 centres), and 210 patients in the BPA treatment group (22 centres). The patient flowchart is shown in Fig. 1.

The baseline characteristics of the participants are summarized in Table 1. The mean age of the cohort was 77 ± 5 years, with 48 males (20.4 %). Patients in the BPA treatment group were significantly older than those in the PEA treatment group (75 ± 3 vs. 77 ± 5, *p* = 0.003). Among the 25 patients in the PEA treatment group, three (12.0 %) were in their 80 s, compared to 65 (47.6 %) of the 210 patients in the BPA treatment group. The two groups were comparable in terms of demographic characteristics, disease severity indices, comorbidities, and medical history. Additionally, BPA was added during follow-up in 11 patients (44.0 %) in the PEA treatment group after initial surgical treatment.

**Table 1:** Baseline characteristics of enrolled patients.

	All patients (n = 235)	PEA treatment group (N = 25)	BPA treatment group (N = 210)	*p*-value
**Demographics**				
Age (years)	77 ± 5	75 ± 3	77 ± 5	0.003
Male, n (%)	48 (20.4)	7 (28.0)	41 (19.5)	0.227
Height (cm)	153.1 ± 8.3	154.5 ± 9.7	153.0 ± 8.1	0.394
Body weight (kg)	52.4 ± 9.7	55.7 ± 11.6	52.0 ± 9.4	0.069
Body mass index	22.3 ± 3.3	23.2 ± 3.3	22.2 ± 3.2	0.132

**Indices of disease severity**				
WHO FC I/II/III/IV, n	2/85/140/8	0/10/15/0	2/75/125/8	0.744
6MWD (m)	319 ± 107	318 ± 122	319 ± 104	0.959
Mean PAP (mmHg)	38 ± 9	38 ± 7	38 ± 10	0.645
PVR (dyn/s/cm^5^)	730 ± 389	682 ± 265	736 ± 401	0.518
CI (L/min/m^2^)	2.5 ± 0.7	2.6 ± 0.6	2.5 ± 0.7	0.513
BNP median (IQR) (pg/mL)	76 (30, 190)	89 (37, 266)	74 (29,183)	0.512
SvO_2_ (%)	63 ± 8	63 ± 6	64 ± 8	0.494
eGFR (mL/min/1.73 m^2^)	58 ± 14.6	60 ± 16	58 ± 14	0.380

**Comorbidities/past medical histories**				
History of acute VTE, n (%)	101 (43.0)	9 (36.0)	92 (43.8)	0.300
Intravenous device, n (%)	4 (1.7)	1 (4.0)	3 (1.4)	0.364
Active cancer/history of cancer, n (%)	24 (10.2)	2 (8.0)	22 (10.5)	0.516
Varicose veins, n (%)	11 (4.7)	0 (0.0)	11 (5.2)	0.282
Thyroid disease, n (%)	9 (3.8)	1 (4.0)	8 (3.8)	0.643
COPD/ILD, n (%)	13 (5.5)	1 (4.0)	12 (5.7)	0.567
Hypercoagulable disorder	10 (4.3)	3 (12.0)	7 (3.3)	0.077
Hemiplegia/paraplegia, n (%)	3 (1.3)	0 (0.0)	3 (1.4)	0.713
Inflammatory bowel disease, n (%)	2 (0.9)	1 (4.0)	1 (0.5)	0.202
History of stroke/TIA, n (%)	13 (5.5)	1 (4.0)	12 (5.7)	0.587
Ovarian/uterine disease, n (%)	20 (8.5)	1 (4.0)	19 (9.0)	0.344
Use of antipsychotics, n (%)	10 (4.3)	1 (4.0)	9 (4.3)	0.712
Use of prednisolone, n (%)	3 (1.3)	0 (0.0)	3 (1.4)	0.713

Values are presented as mean ± SD, n (%), or median (Q1, Q3). Comparison between BPA and PEA treatment groups, *p*<0.05 indicates statistical significance.

BNP, brain natriuretic peptide; BPA, balloon pulmonary angioplasty; CI, cardiac index; COPD, chronic obstructive pulmonary disease; eGFR, estimated glomerular filtration rate; ILD, interstitial lung disease; IQR, interquartile range; 6MWD, 6-minute walk distance; PAP, pulmonary artery pressure; PEA, pulmonary endarterectomy; PVR, pulmonary vascular resistance; SvO2, mixed venous oxygen saturation; TIA, transient ischemic attack; VTE, venous thromboembolism; WHO FC, World Health Organization functional class.

### 3.2 Medications at baseline and follow-up

At baseline, warfarin was used in 12 patients (48.0 %) in the PEA treatment group and 73 (34.8 %) in the BPA treatment group (*p* = 0.193), while direct oral anticoagulants (DOACs) were used in 12 (48.0 %) and 133 (63.3 %) patients, respectively (*p* = 0.136). The use of rivaroxaban was significantly lower in the treatment PEA group (1 [4.0 %]) compared to the BPA treatment group (42 [20.0 %], *p* = 0.035). At follow-up, warfarin was used in 11 patients (44.0 %) in the PEA treatment group and 59 (28.1 %) in the BPA treatment group (*p* = 0.100), while DOACs were used in 14 (66.0 %) and 149 (70.9 %) patients, respectively (*p* = 0.125). Changes in the use of warfarin and DOACs from baseline to follow-up were statistically significant only in the BPA treatment group (*p* = 0.009 and *p* = 0.007, respectively). Regarding pulmonary vasodilators, their use at baseline was observed in 10 patients (40.0 %) in the PEA treatment group and 102 (48.6 %) in the BPA treatment group (*p* = 0.417), and at follow-up in 13 (52.0 %) and 130 (61.9 %) patients, respectively (*p* = 0.337). The use of soluble guanylate cyclase stimulators, such as riociguat, was 10 (40.0 %) at baseline and 13 (52.0 %) at follow-up in the PEA treatment group, with no statistically significant change (p = 0.549). In contrast, the use significantly increased from 86 (41.0 %) to 117 (55.7 %) in the BPA treatment group (p < 0.001). Additionally, the use of prostaglandin I_2_ receptor agonists, such as selexipag, was zero (0 %) at baseline and 3 (12.0 %) at follow-up in the PEA treatment group (*p* = 0.25), and significantly increased from 15 (7.1 %) to 39 (18.6 %) in the BPA treatment group (*p* < 0.001). Detailed medication data for both groups are provided in Table 2 and Supplementary Table S1.

**Table 2:** Medications at baseline and follow-up.

	All patients (n = 235)	PEA treatment group (N = 25)	BPA treatment group (N = 210)	*p*-value
**At baseline**				
**Anticoagulants**				
Warfarin	85 (36.2)	12 (48.0)	73 (34.8)	0.193
DOACs	145 (61.7)	12 (48.0)	133 (63.3)	0.136
Dabigatran	2 (0.9)	0 (0)	2 (1.0)	0.798
Apixaban	56 (23.8)	4 (16.0)	52 (24.8)	0.331
Rivaroxaban	43 (18.3)	1 (4.0)	42 (20.0)	0.035
Edoxaban	44 (18.7)	7 (28.0)	37 (17.6)	0.161
No oral anticoagulants	5 (2.1)	1 (4.0)	4 (1.9)	0.433

**Pulmonary vasodilators**				
Any pulmonary vasodilator	112 (47.7)	10 (40.0)	102 (48.6)	0.417
Endothelin receptor antagonists	10 (4.3)	1 (4.0)	9 (4.3)	0.947
PDE-5 inhibitors/sGC stimulators	98 (41.7)	10 (40.0)	88 (41.9)	0.855
Riociguat	96 (40.9)	10 (40.0)	86 (41.0)	0.927
Prostacyclin analog/PGI2 receptor agonists	19 (8.1)	2 (8.0)	17 (8.1)	0.987
Selexipag	15 (6.4)	0 (0)	15 (7.1)	0.175

**At follow-up**				
**Anticoagulants**				
Warfarin	70 (29.8)	11 (44.0)	59 (28.1)	0.100
DOACs	163 (69.4)	14 (66.0)	149 (70.9)	0.125
Dabigatran	2 (0.9)	0 (0)	2 (1.0)	0.798
Apixaban	55 (23.4)	2 (8.0)	53 (25.2)	0.054
Rivaroxaban	56 (23.8)	7 (28.0)	49 (23.3)	0.605
Edoxaban	50 (21.3)	5 (20.0)	45 (21.4)	0.869
No oral anticoagulants	2 (0.9)	0 (0)	2 (1.0)	0.798

**Pulmonary vasodilators**				
Any pulmonary vasodilator	143 (60.9)	13 (52.0)	130 (61.9)	0.337
Endothelin receptor antagonists	0 (0)	0 (0)	0 (0)	NA
PDE-5 inhibitors/sGC stimulators	130 (55.3)	13 (52.0)	117 (55.7)	0.724
Riociguat	130 (55.3)	13 (52.0)	117 (55.7)	0.724
Prostacyclin analog/PGI2 receptor agonists	42 (17.9)	3 (12.0)	39 (18.6)	0.309
Selexipag	42 (17.9)	3 (12.0)	39 (18.6)	0.309

Values are presented as n (%). Comparison between BPA and PEA treatment groups, *p*<0.05 indicates statistical significance.

BPA, balloon pulmonary angioplasty; DOACs, direct oral anticoagulants; NA, not applicable; PDE5, phosphodiesterase-5; PEA, pulmonary endarterectomy; PGI2, prostaglandin I2; sGC, soluble guanylate cyclase.

### 3.3 Changes in disease severity indices between baseline and follow-up

**Fig. 2:**
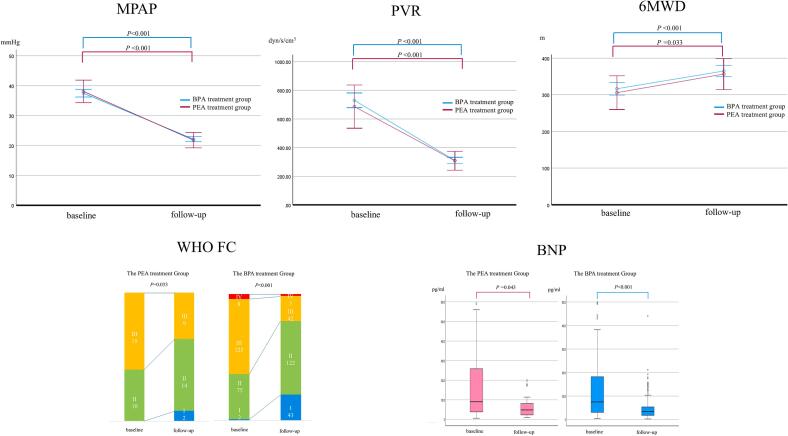
**Changes in the indices of disease severity from baseline to follow-up.** Comparisons between baseline and follow-up were performed using a paired samples *t*-test for parametric variables and the Wilcoxon's signed-rank test for nonparametric variables. BPA, balloon pulmonary angioplasty; BNP, brain natriuretic peptide; 6MWD, six-minute walk distance; MPAP, mean pulmonary artery pressure; PEA, pulmonary endarterectomy; PVR, pulmonary vascular resistance; WHO FC, World Health Organization functional class

Table 3 and Fig. 2 illustrate the change from baseline to follow-up. The median follow-up period across all patients was 723 days (range: 397–1188 days). In the PEA treatment group, the follow-up period was 952 days (range: 731–1316 days), whereas in the BPA group, it was 790 days (range: 378–1165 days) (*p* = 0.110).

**Table 3:** Comparison of changes in indices of disease severity.

	PEA treatment group				BPA treatment group				Difference between changes**		
	Baseline	Follow-up	Change	*p*-value	Baseline	Follow-up	Change	*p*-value*	Mean (95 % CI)	*p*-value	Adjusted *p*-value**
WHO FC I/II n (%)	10 (40.0)	16 (64.0)	6 (24.0)	0.033	77 (36.7)	165 (78.6)	88 (41.9)	<0.001		0.087	0.035
6MWD (m)	306 ± 129	356 ± 92	51 ± 99	0.033	317 ± 100	365 ± 96	48 ± 86	<0.001	2 (−33 to 38)	0.903	0.380
Mean PAP (mmHg)	38 ± 7	22 ± 7	−15 ± 9	<0.001	38 ± 10	22 ± 6	−15 ± 10	<0.001	0.4 (−2 to 3)	0.747	0.997
PVR (dyn/s/cm^5^)	686 ± 276	308 ± 200	−365 ± 285	<0.001	730 ± 383	310 ± 157	−430 ± 384	<0.001	−42 (−195 to 111)	0.589	0.667
CI (L/min/m^2^)	2.6 ± 0.6	2.5 ± 0.5	−0.1 ± 0.6	0.660	2.5 ± 0.7	2.6 ± 0.6	0.2 ± 0.6	<0.001	0.2 (−0.1 to 0.4)	0.140	0.166
BNP median (IQR) (pg/mL)	89 (37, 266)	48 (21, 83)	−128 ± 228	0.043	75 (30, 183)	34 (18, 53)	−127 ± 286	<0.001	3 (−56 to 61)	0.932	0.990
SvO_2_ (%)	63 ± 6	67 ± 11	5 ± 12	0.077	64 ± 8	69 ± 6	5 ± 8	<0.001	2 (−1 to 5)	0.187	0.065
eGFR (mL/min/1.73 m^2^)	60 ± 17	58 ± 16	−2 ± 12	0.374	57 ± 14	58 ± 14	0.3 ± 11	0.715	2 (−3 to 6)	0.445	0.403

Values are presented as mean±SD, or median (Q1, Q3).

BNP, brain natriuretic peptide; BPA, balloon pulmonary angioplasty; CI, cardiac index; eGFR, estimated glomerular filtration rate; IQR, interquartile range; 6MWD, 6-minute walk distance; PAP, pulmonary artery pressure; PEA, pulmonary endarterectomy; PVR, pulmonary vascular resistance; SvO2, mixed venous oxygen saturation; WHO FC, World Health Organization functional class.

* Comparisons of data between baseline and follow-up were tested using a paired samples *t*-test for parametric variables and Wilcoxon's signed-rank test for nonparametric variables.

** Differences between changes in the PEA treatment group and the BPA treatment group were tested using analysis of covariance (ANCOVA) for continuous variables and a logistic regression model for categorical variables, with age, sex, and body mass index as covariates.

In the PEA treatment group, WHO FC distribution significantly improved from I/II/III/IV: 0/10/15/0 at baseline to 2/14/9/0 at follow-up (p = 0.033). and 6MWD increased from 306 ± 129 m to 357 ± 92 m (*p* = 0.033). Hemodynamic parameters showed a reduction in mean PAP from 38 ± 7 mmHg to 22 ± 7 mmHg (*p* < 0.001), and PVR decreased from 686 ± 276 dyn/s/cm^5^ to 308 ± 200 dyn/s/cm^5^ (*p* < 0.001). Regarding blood test data, BNP levels declined from a median of 89 pg/mL (interquartile range: 37–266 pg/mL) to 48 pg/mL (interquartile range:21–83 pg/mL) (*p* = 0.043).

In the BPA treatment group, WHO FC distribution significantly improved from I/II/III/IV: 2/75/125/8 at baseline to 43/122/42/3 at follow-up (p < 0.001). and 6MWD increased from 317 ± 100 m to 365 ± 96 m (*p* < 0.001). Hemodynamic parameters showed a decrease in mean PAP from 38 ± 10 mmHg to 22 ± 6 mmHg (*p* < 0.001), and PVR from 730 ± 383 dyn/s/cm^5^ to 310 ± 157 dyn/s/cm^5^ (*p* < 0.001). BNP levels also decreased from a median of 75 pg/mL (interquartile range: 30–183 pg/mL) to 34 pg/mL (interquartile range: 18–53 pg/mL) (*p* < 0.001).

In the PEA treatment group, neither CI nor SvO_2_ increased significantly, with CI measuring 2.6 ± 0.6 L/min/m^2^ at baseline compared to 2.5 ± 0.5 L/min/m^2^ at follow-up (*p* = 0.660), and SvO_2_ measuring 63 ± 6 % compared to 67 ± 11 %, respectively (*p* = 0.077). Conversely, in the BPA treatment group, both CI and SvO_2_ improved significantly, with CI increasing from 2.5 ± 0.7 L/min/m^2^ to 2.6 ± 0.6 L/min/m^2^ (*p* < 0.001) and SvO_2_ from 64 ± 8 % to 69 ± 6 % (*p* < 0.001). Renal function, such as the eGFR, did not show significant changes in either group: in the PEA treatment group, eGFR changed from 60 ± 17 mL/min/1.73  m^2^ to 58 ± 16 mL/min/1.73  m^2^ (p = 0.374); in the BPA treatment group, it changed from 57 ± 14 to 58 ± 14 mL/min/1.73  m^2^ (*p* = 0.715).

After adjustment for potential covariates (*i.e.*, age, sex and body mass index), the changes in 6MWD, mean PAP, PVR, and CI were not significantly different between the PEA and BPA treatment groups, with between-group differences of 2 m (95 % CI: −33 to 38, adjusted *p* = 0.380), 0.4 mmHg (95 % CI: −2 to 3, adjusted *p* = 0.997), −42 dyn·s·cm^−5^ (95 % CI: −195 to 111, adjusted *p* = 0.667), and 0.2 L/min/m^2^ (95 % CI: −0.1 to 0.4, adjusted *p* = 0.166), respectively. In contrast, after covariate adjustment, improvement in WHO FC was significantly greater in the BPA group than in the PEA group (adjusted *p* = 0.035).

### 3.4 Adverse events during follow-up

During the follow-up period, the adverse events included a total of five all-cause deaths (2.1 %), one rescue intervention (0.4 %), three cases of pH recurrence (1.3 %), nine cases of major bleeding (3.8 %), and three cases of minor bleeding (1.3 %), indicating a low incidence rate. Notably, all deaths occurred during the follow-up, with none reported during the perioperative period or hospitalization for treatment.

In total, one all-cause death (4.0 %), one case of pH exacerbation (4.0 %), and two cases of major bleeding (8.0 %) were observed in the PEA treatment group. The BPA treatment group reported four all-cause deaths (1.9 %), one rescue intervention (0.4 %), two cases of pH exacerbation (1.0 %), seven cases of major bleeding (3.3 %), and three cases of minor bleeding (1.4 %). The associations between treatment strategies and adverse events were analysed using a multivariable logistic regression model, adjusting for age, sex, and body mass index as covariates; however, no statistically significant associations were observed. No cases of lung transplantation, prostaglandin I_2_ injections, or recurrence of VTE were observed in either group. Detailed adverse event data for both groups are summarized in Table 4.

**Table 4:** Adverse events at follow-up.

	All patients (n = 235)	PEA treatment group (n = 25)	BPA treatment group (n = 210)	*p*-value	Adjusted *p*-value*
**Adverse events**					
All adverse events	19 (8.1)	3 (12.0)	16 (7.6)	0.323	0.187
All-cause death	5 (2.1)	1 (4.0)	4 (1.9)	0.433	0.248
Lung transplantation	0 (0)	0 (0)	0 (0)	NA	NA
Use of PGI2 infusion	0 (0)	0 (0)	0 (0)	NA	NA
Rescue intervention such as BPA and PEA	1 (0.4)	0 (0)	1 (0.5)	0.894	0.998
Worsening PH	3 (1.3)	1 (4.0)	2 (1.0)	0.287	0.220
Major bleeding	9 (3.8)	2 (8.0)	7 (3.3)	0.246	0.164
Minor bleeding	3 (1.3)	0 (0)	3 (1.4)	0.713	0.998
Recurrence of VTE	0 (0)	0 (0)	0 (0)	NA	NA

Values are presented as n (%).

BPA, balloon pulmonary angioplasty; NA, not applicable; PEA, pulmonary endarterectomy; PVR, pulmonary vascular resistance; PGI2, prostaglandin I2; PH, pulmonary hypertension.

* The associations of treatment strategies with adverse events were analyzed using a multivariable logistic regression model after adjusting for age, sex, and body mass index as covariates.

The Kaplan–Meier cumulative curves for all adverse events and all causes of death are presented in Supplementary Fig. S1. The log-rank test results indicated no significant differences (*p* = 0.616 for all adverse events; *p* = 0.564 for all-cause death).

## 4 Discussion

In the present analysis of patients aged 70 years or older who underwent PEA or BPA, our multivariable models showed comparable improvements in hemodynamic parameters, exercise capacity, and biomarkers such as BNP, with similar survival outcomes between the two groups. However, the subjective measure of symptoms, represented by WHO FC, was more favourable among patients who received BPA than among those who received PEA. Notably, patients in the BPA treatment group tended to be older than those in the PEA group; specifically, 65 patients (47.6 %) in the BPA treatment group were in their 80 s, in contrast to only three patients (12.0 %) in the PEA treatment group who were in this age group.

PEA remains the standard treatment for CTEPH; it is a highly invasive procedure that requires a median sternotomy and deep hypothermic cardiopulmonary bypass, often below 20 °C [11]. Although Berman et al. argue that age alone should not preclude PEA, they reported that advanced age is associated with extended ICU stays, prolonged hospitalizations, and increased healthcare costs [12]. Newnham et al. reported that although patients aged 80 years or older who underwent PEA experienced similar treatment efficacy to those under 80 years of age, their hospital stays were longer, and recovery was slower [13]. Moreover, although PEA can achieve favourable hemodynamic and functional outcomes even in older patients, the prolonged recovery and extended hospitalization associated with this invasive procedure may contribute to increased frailty and delayed improvement in subjective symptoms, particularly among older patients. These factors may be among several contributing factors that could explain smaller improvement in WHO FC observed in patients who underwent PEA, despite comparable hemodynamic efficacy. Conversely, research on BPA in adults aged 80 years or older has demonstrated its efficacy for CTEPH in this age group [14]. Thus, the high invasiveness of PEA may influence the decision-making process regarding treatment options for older adults.

In another previous study on CTEPH that included patients across all age groups, the average age of patients who underwent PEA was 60 years, and for those undergoing BPA, it was 66 years [15]. A similar trend is observed in Japan, where patients receiving PEA average 63 years of age, and those undergoing BPA average 67 years of age [16]. Aging is advancing globally, with Japan experiencing an especially rapid demographic shift. As a result, the age distribution of patients with CTEPH has changed, with diagnoses increasingly occurring in individuals in their 70 s, and cases in those aged over 80 years becoming increasingly common. In this study, 34.7 % of patients who underwent PEA and 50.6 % of those who underwent BPA were aged 70 years or older. This trend highlights the growing importance of considering invasive treatments for older adults with CTEPH in the future.

In this study, 11 of 25 patients (44.0 %) in the PEA treatment group underwent planned BPA during the follow-up period. Residual PH after PEA is known to affect approximately 25 % of cases, with older patients with CTEPH showing a higher likelihood of residual PH [17,18]. Previous studies have demonstrated that BPA for residual PH post-PEA can improve symptoms and hemodynamics [19,20]. However, a study suggests that in patients aged 70 years or older, symptoms can improve, and prognosis may remain favourable even if BPA is discontinued, owing to frailty and comorbidities [14]. Given that BPA can carry risks such as pulmonary hemorrhage, it is essential to consider whether treatment goals in older adults should be as intensive as those for younger patients [21,22].

Regarding anticoagulant therapy, guidelines recommend lifelong continuous use for patients with CTEPH, with vitamin K antagonists (VKAs) traditionally considered the preferred option [5,23]. Some reports have indicated comparable bleeding rates between VKAs and DOACs in patients with CTEPH; however, recurrent VTE rates were higher in those receiving DOACs [24,25]. A multicenter, single-blind, randomized trial in Japan demonstrated that edoxaban was non-inferior to warfarin in preventing the worsening of pH and also reported lower rates of VTE recurrence and bleeding events [26]. In the present study, 145 patients (61.7 %), including 12 (48.0 %) in the PEA treatment group and 133 (63.3 %) in the BPA treatment group, received DOACs at baseline. Furthermore, during follow-up, the use of DOACs increased significantly in the BPA treatment group. Given that the BPA group was older, it is possible that DOACs were preferred due to their lower risk of bleeding. No recurrent VTE events were observed in either group, and bleeding events (both major and minor) were infrequent, with two patients (8.0 %) in the PEA treatment group and 10 patients (4.8 %) in the BPA treatment group. It has been reported that in older patients with atrial fibrillation or VTE, the use of DOACs results in fewer bleeding complications than the use of VKAs. Although current evidence on the use of DOACs in patients with CTEPH remains limited, including the present study, their use is not yet actively promoted; nevertheless, DOACs may become an increasingly preferred anticoagulant for older patients with CTEPH in the future [27,28].

Pulmonary vasodilators, such as riociguat and selexipag, have been reported to effectively improve exercise tolerance and symptoms in patients with CTEPH [29,30]. In the present study, despite improvements in WHO FC and hemodynamics in both groups, the usage rates of these medications remained high after treatment. This may be partly due to the limited number of patients achieving WHO FC I, suggesting the persistence of subjective symptoms or physician caution in discontinuing therapy. Furthermore, there was no significant difference in the usage rates of these medications between the two groups at baseline and follow-up. However, the increase in usage rates during the observation period was statistically significant in the BPA treatment group but not in the PEA treatment group. Although the proportion of older adults was higher in the BPA treatment group than in the PEA treatment group, this does not necessarily indicate that more aggressive pharmacotherapy was intentionally applied. It is possible that patient characteristics and institutional treatment strategies collectively influenced the use of pulmonary vasodilators. This, in turn, may be associated with the greater improvement in WHO FC observed in the BPA treatment group. Therefore, the effect of BPA on functional class improvement should be interpreted with caution, as it may be partially attributable to concomitant pharmacotherapy.

In this study, CI remained almost unchanged in the PEA group, whereas it significantly increased in the BPA treatment group during the follow-up period. This finding is in contrast to previous reports, in which CI typically increases after PEA but does not increase substantially after BPA. This discrepancy may also be influenced by the use of pulmonary vasodilators in the BPA treatment group.

### 4.1 Limitations

This study has several limitations. First, as this study was based on registry data, the decision to administer PEA or BPA was at the discretion of the treating physicians, and the same applies to pharmacological therapy. Given that CTEPH is a rare disease, there are differences in treatment experience, the number of BPA procedures, and pharmacological treatment before and after PEA or BPA across institutions. In addition, patient backgrounds, such as age, and variations in CTEPH severity and lesion complexity may lead to differences in treatment goals and outcomes. Second, while this registry records whether PEA or BPA procedures were performed, it lacks details on procedural specifics, including procedure duration, devices used, intraoperative complications, and complication severity. In addition, the timing of initiation and discontinuation of pharmacological therapies, changes in pulmonary vasodilator dosage, and the management quality of VKAs during the treatment period are not captured. These factors may not only influence overall treatment outcomes and long-term prognosis but may also account for differences in outcomes among institutions. Furthermore, the exact timing of baseline and follow-up data collection varied among centres and patients due to the registry-based design; therefore, the timing relative to diagnosis or intervention could not be standardized. This limitation may affect the interpretation of longitudinal changes and treatment effects. Further investigation incorporating these variables is warranted. Third, the current treatment landscape in Japan, where BPA is more commonly performed than PEA and combination drug therapy is frequently used, may also influence the results, potentially limiting the generalizability of the findings. Fourth, the data were primarily obtained from Japanese patients; hence, it remains unclear whether similar outcomes would be applicable to other ethnic groups. Fifth, symptoms, quality of life, and treatment satisfaction were not assessed using patient-reported outcomes. To accurately capture the patient's perspective and enhance the clinical significance of the findings, future investigations should consider incorporating patient-reported outcomes.

### 4.2 Conclusions

Among adults aged 70 years or older with CTEPH, exercise capacity, hemodynamic parameters, and clinical outcomes appeared generally comparable between those who underwent PEA and those who underwent BPA. However, subjective improvement in WHO FC was more frequently observed in the BPA group. Additionally, patients who underwent BPA were generally older than those who underwent PEA.

## CRediT authorship contribution statement

**Jun Yamashita:** Writing – review & editing, Writing – original draft, Visualization, Validation, Methodology, Investigation, Formal analysis, Conceptualization. **Hitoshi Ogino:** Writing – review & editing, Supervision, Methodology, Investigation, Conceptualization. **Kohei Masaki:** Writing – original draft, Software, Project administration, Methodology, Investigation, Data curation, Conceptualization. **Yu Taniguchi:** Writing – review & editing, Investigation, Conceptualization. **Shiro Adachi:** Writing – review & editing, Methodology, Investigation. **Takumi Inami:** Writing – review & editing, Methodology, Investigation, Conceptualization. **Kazuya Hosokawa:** Writing – review & editing, Validation, Software, Resources, Project administration, Methodology, Investigation, Funding acquisition, Data curation. **Ichizo Tsujino:** Writing – review & editing, Investigation. **Nobuhiro Yaoita:** Writing – review & editing, Investigation. **Masaru Hatano:** Writing – review & editing, Investigation. **Nobutaka Ikeda:** Writing – review & editing, Investigation, Conceptualization. **Nobuhiro Tanabe:** Writing – review & editing, Supervision, Investigation. **Hiroto Shimokawahara:** Writing – review & editing, Investigation, Conceptualization. **Kayoko Kubota:** Writing – review & editing, Investigation. **Ayako Shigeta:** Writing – review & editing, Investigation. **Koshin Horimoto:** Writing – review & editing, Investigation. **Yoshito Ogihara:** Writing – review & editing, Investigation. **Yoshihiro Dohi:** Writing – review & editing, Investigation. **Takahiro Hiraide:** Writing – review & editing, Investigation. **Takashi Kawakami:** Writing – review & editing, Investigation. **Hidekazu Ikemiyagi:** Writing – review & editing, Investigation. **Yuichi Tamura:** Writing – review & editing, Supervision, Investigation, Conceptualization. **Yoshihiro Fukumoto:** Writing – review & editing, Supervision, Methodology, Conceptualization. **Kohtaro Abe:** Writing – review & editing, Visualization, Validation, Supervision, Resources, Project administration, Methodology, Investigation, Funding acquisition, Formal analysis, Data curation, Conceptualization.

## Funding

This work was supported by the Japan Agency for Medical Research and Development (grant numbers: JP20ek0109371, JP19lk0201102, JP22lk0201125, and JP19lk1601003). The funding body had no role in the design and conduct of the study; collection, management, analysis, and interpretation of the data; preparation, review, or approval of the manuscript; or decision to submit the manuscript for publication.

## Declaration of competing interest

The authors declare the following financial interests/personal relationships which may be considered as potential competing interests: Jun Yamashita reports grants from Abbott Medical Japan and WIN International and personal fees from Kaneka Medix, Boston Scientific Japan, Nihon Kohden, Nippon Shinyaku, Mallinckrodt Pharma, AstraZeneca, and Kowa Company outside the submitted work. Hitoshi Ogino reports consulting fees from Terumo, Japan Lifeline, and Century Medical outside the submitted work. Yu Taniguchi reported grants from Janssen Pharmaceutical and Nippon Shinyaku, as well as personal fees from Janssen Pharmaceutical and Nippon Shinyaku, outside the submitted work. Shiro Adachi reports grants from Nippon Shinyaku, Janssen Pharmaceutical Japan, MSD, and Mochida Pharmaceutical and personal fees from Nippon Shinyaku, Bayer Yakuhin, and Janssen Pharmaceutical Japan. Takumi Inami reports personal fees from Janssen Pharmaceutical and Bayer Yakuhin outside the submitted work. Kazuya Hosokawa reports personal fees from Janssen Pharmaceutical, Bayer Yakuhin, Nippon Shinyaku, and Pfizer outside the submitted work. Ichizo Tsujino reports personal fees from Janssen Pharmaceutical and Nippon Shinyaku and affiliation with a division supported by endowments from Nippon Shinyaku and Nippon Boehringer Ingelheim, Mochida Pharmaceutical, Kaneka, Takeyama, and the Medical System Network outside the submitted work. Nobuhiro Yaoita reports personal fees from Bayer Yakuhin and Konica Minolta outside the submitted work. Nobutaka Ikeda reports personal fees from Janssen Pharmaceutical, Bayer Yakuhin, Nippon Shinyaku, Daiichi Sankyo, and Bristol-Myers Squibb outside the submitted work. Nobuhiro Tanabe reports personal fees from Janssen Pharmaceutical, Bayer Yakuhin, and Nippon Shinyaku outside the submitted work. Hiroto Shimokawahara reports a grant from Bayer Yakuhin and personal fees from Actelion Pharmaceuticals Japan, Bayer Yakuhin, and Nippon Shinyaku outside the submitted work.Kayoko Kubota reports personal fees from Janssen Pharmaceutical and Nippon Shinyaku outside the submitted work. Ayako Shigeta reports personal fees from Janssen Pharmaceutical, Bayer Yakuhin, Nippon Shinyaku, Mochida Pharmaceutical, and Merck Sharp & Dohme outside the submitted work. Yoshito Ogihara reports personal fees from Janssen Pharmaceutical, Bayer Yakuhin, and Nippon Shinyaku, as well as grants from Bayer Yakuhin outside the submitted work. Yoshihiro Dohi reports personal fees from Janssen Pharmaceutical, Bayer Yakuhin, and Nippon Shinyaku outside the submitted work.Takahiro Hiraide reports grants from Janssen Pharmaceutical and Nippon Shinyaku and personal fees from Janssen Pharmaceutical, Mochida Pharmaceutical, and Nippon Shinyaku, outside the submitted work. Takashi Kawakami reports personal fees from Kaneka Medix, Abbott Medical Japan, and ACIST Japan outside the submitted work. Yuichi Tamura reports grants from Bayer Yakuhin, Nippon Shinyaku, and Mochida Pharmaceutical and personal fees from Bayer Yakuhin, Nippon Shinyaku, Daiichi Sankyo, and Janssen Pharmaceutical outside the submitted work. Yoshihiro Fukumoto reports grants from Janssen Pharmaceutical and MSD K.K. and lecture fees from Bayer Yakuhin, Nippon Shinyaku, Mochida Pharmaceutical, Daiichi Sankyo, MSD K.K., and Janssen Pharmaceutical outside the submitted work and personal fees from Bayer Yakuhin, Nippon Shinyaku, Daiichi Sankyo, and Janssen Pharmaceutical outside the submitted work. Kohtaro Abe reports grants from Konica Minolta and Daiichi Sankyo outside the submitted work. Kohei Masaki , Koshin Horimoto , Masaru Hatano , and Hidekazu Ikemiyagi report no conflicts of interest.

## Glossary

BNP: brain natriuretic peptide
BPA: balloon pulmonary angioplasty
CI: cardiac index
COPD: chronic obstructive pulmonary disease
DOACs: direct oral anticoagulants
eGFR: estimated glomerular filtration rate
ILD: interstitial lung disease
6MWD: 6-minute walk distance
PAP: pulmonary artery pressure
PDE5: phosphodiesterase-5
PEA: pulmonary endarterectomy
PVR: pulmonary vascular resistance
SvO_2_: mixed venous oxygen saturation
TIA: transient ischemic attack
VTE: venous thromboembolism
WHO FC: World Health Organization functional class
PROs: patient-reported outcomes
