# Supplementary figures and images for: Effects of pulmonary endarterectomy and balloon pulmonary angioplasty in older adults with chronic thromboembolic pulmonary hypertension: A sub-analysis of the CTEPH AC registry
Source: Int J Cardiol Heart Vasc. 2025 Jul 12;60:101751. doi: 10.1016/j.ijcha.2025.101751 (PMC12274849; doi:10.1016/j.ijcha.2025.101751)

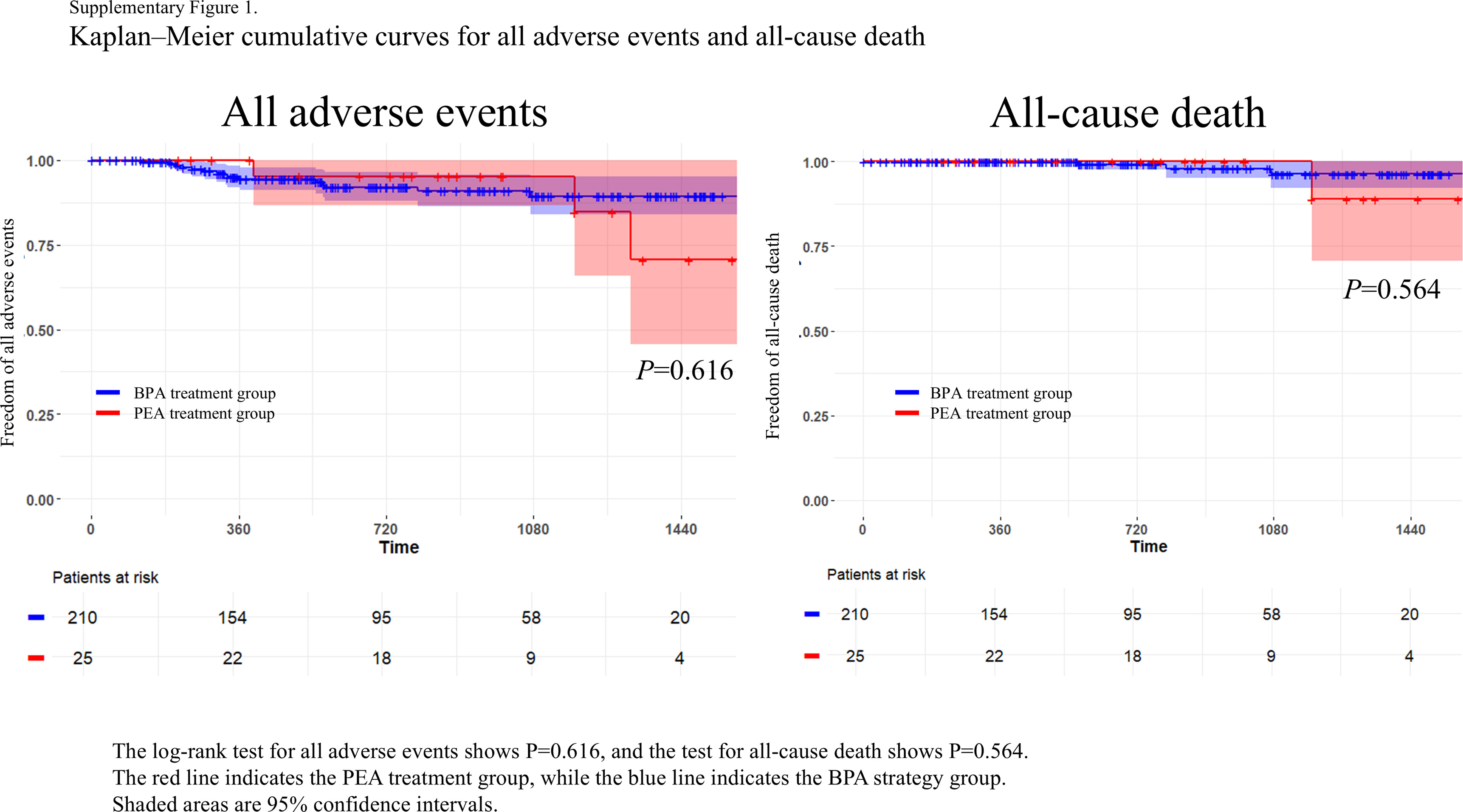

Supplement: Supplementary Figure 1 [file mmc1.jpg]
