# Supplementary material for: Effects of pulmonary endarterectomy and balloon pulmonary angioplasty in older adults with chronic thromboembolic pulmonary hypertension: A sub-analysis of the CTEPH AC registry
Source: Int J Cardiol Heart Vasc. 2025 Jul 12;60:101751. doi: 10.1016/j.ijcha.2025.101751 (PMC12274849; doi:10.1016/j.ijcha.2025.101751)
Supplement: Supplementary Data 2 [file mmc2.docx]

**Supplementary Table 1.**

**Changes in medications** **from baseline to follow-up**

|  | **PEA treatment group**  **(N=25)** | | | **BPA treatment group**  **(N=210)** | | |
| --- | --- | --- | --- | --- | --- | --- |
|  | **Baseline** | **Follow-up** | ***p*-value*** | **Baseline** | **Follow-up** | ***p*-value*** |
| **Anticoagulants** |  |  |  |  |  |  |
| Warfarin, n (%) | 12 (48.0) | 11 (44.0) | 1.0 | 73 (34.8) | 59 (28.1) | 0.009 |
| DOACs, n (%) | 12 (48.0) | 14 (66.0) | 0.754 | 133 (63.3) | 149 (70.9) | 0.007 |
| **Pulmonary vasodilators** |  |  |  |  |  |  |
| Any pulmonary vasodilator, n (%) | 10 (40.0) | 13 (52.0) | 0.549 | 102 (48.6) | 130 (61.9) | 0.001 |
| Endothelin receptor antagonists | 1 (4.0) | 0 (0) | 1.0 | 9 (4.3) | 0 (0) | 0.003 |
| PDE-5 inhibitors/sGC stimulators | 10 (40.0) | 13 (52.0) | 0.549 | 88 (41.9) | 117 (55.7) | <0.001 |
| Riociguat | 10 (40.0) | 13 (52.0) | 0.549 | 86 (41.0) | 117 (55.7) | <0.001 |
| Prostacyclin analog/PGI2 receptor agonists | 2 (8.0) | 3 (12.0) | 1.0 | 17 (8.1) | 39 (18.6) | <0.001 |
| Selexipag | 0 (0) | 3 (12.0) | 0.25 | 15 (7.1) | 39 (18.6) | <0.001 |

_Values are presented as n (%). Comparison between BPA and PEA treatment groups,_ *_p_*_<0.05 statistically significant._

_BPA, balloon pulmonary angioplasty; DOACs, direct oral anticoagulants; PDE5, phosphodiesterase-5; PEA, pulmonary endarterectomy; PGI2, prostaglandin I2; sGC, soluble guanylate cyclase_

_*_ _Comparisons of data between baseline and follow-up were conducted using McNemar’s test._
